# Pyoverdine-Mediated Killing of *Caenorhabditis elegans* by *Pseudomonas syringae* MB03 and the Role of Iron in Its Pathogenicity

**DOI:** 10.3390/ijms21062198

**Published:** 2020-03-22

**Authors:** Anum Bashir, Tian Tian, Xun Yu, Cui Meng, Muhammad Ali, Lin Li

**Affiliations:** 1 State Key Laboratory of Agricultural Microbiology, Huazhong Agricultural University, Wuhan 430070, China; anumbashir@webmail.hzau.edu.cn (A.B.); 18155377062@163.com (T.T.); yuxun@webmail.hzau.edu.cn (X.Y.); ali@cuiatd.edu.pk (M.A.); 2 Department of Biotechnology, COMSATS University Islamabad, Abbottabad Campus, Abbottabad 22060, Pakistan

**Keywords:** pyoverdine, iron, *Pseudomonas syringae*, *Caenorhabditiselegans*, pathogenicity, liquid killing assay

## Abstract

The pathogenicity of the common phytopathogenic bacterium *Pseudomonas syringae* toward *Caenorhabditis elegans* has been recently demonstrated. However, the major virulence factors involved in this interaction remain unknown. In this study, we investigated the nematocidal activity of *P. syringae* against *C. elegans* under iron-sufficient/limited conditions, primarily focusing on the role of the ferric chelator pyoverdine in a *P. syringae*–*C. elegans* liquid-based pathogenicity model. Prediction-based analysis of pyoverdine-encoding genes in the genome of the wild-type *P. syringae* strain MB03 revealed that the genes are located in one large cluster. Two non-ribosomal peptide synthetase genes (*pvdD* and *pvdJ*) were disrupted via a Rec/TE recombination system, resulting in mutant strains with abrogated pyoverdine production and attenuated virulence against *C. elegans.* When used alone, pure pyoverdine also showed nematocidal activity. The role of iron used alone or with pyoverdine was further investigated in mutant and MB03-based bioassays. The results indicated that pyoverdine in *P. syringae* MB03 is a robust virulence factor that promotes the killing of *C. elegans*. We speculate that pyoverdine functions as a virulence determinant by capturing environmentally available iron for host bacterial cells, by limiting its availability for *C. elegans* worms, and by regulating and/or activating other intracellular virulence factors that ultimately kills *C. elegans* worms.

## 1. Introduction

Iron is an essential trace element that is used by nearly all microorganisms to maintain normal life activities, and it functions as a cofactor for many oxidoreductases that catalyze redox reactions while also being involved in a broad range of vital biochemical processes, such as electron transfer, oxygen transport, and energy production [1,2]. Although iron is one of the most abundant elements in the Earth’s crust, the soluble ferrous iron (Fe^2+^) that is accessible to bacterial cells is easily oxidized to less soluble ferric (Fe^3+^) oxides or hydroxides under aerobic and physiological pH conditions, leading the concentration of Fe^2+^ to typically be only 10^−9^ to 10^−18^ mol L^-1^ in the environment, which is much lower than the normal concentration required for bacterial cells (10^−5^ to 10^−7^ mol L^−1^) [3,4]. Therefore, bacterial cells are typically in an iron-deficient stress state and the struggle for iron is always an important combat amongst various bacterial species. To counter the problems imposed by their iron dependence, a number of bacteria have evolved different mechanisms to acquire iron [5] One of the most common strategies is the synthesis of ferric chelators known as siderophores, which are capable of taking up ferric iron from the environment [6]

Siderophores are small molecules (1–2 kDa) that are synthesized and secreted by bacterial cells in response to low-iron conditions and have a high specificity and affinity (*K_aff_* > 10^30^ mol L^−1^) for ferric iron [3]. Based on the functional ligands used for iron chelation, siderophores can be generally classified into three types, i.e., catechol, hydroxamate, and α-hydroxycarboxylate types [1]. The dual iron-chelating groups in the ligands catecholate and hydroxamate can generally confer an extremely effective ferric iron-chelating capability. Many siderophores are composed of peptides, which are commonly synthesized by non-ribosomal peptide synthetases (NRPSs) and multi-enzyme systems, whereas other non-polypeptide siderophores are produced by NRPS-independent synthesis pathways [7,8]. Following their biosynthesis, siderophores are secreted across the cell membrane to the extracellular space to capture ferric iron from the environment, resulting in the formation of ferric-siderophore complexes that then bind to the corresponding receptors on the outer membrane (OM) and transport the iron to the cytosol via a cytoplasm-directed ABC (ATP-binding cassette) transporter [9,10]. In addition to iron, siderophores can also uptake and transfer a variety of other metals, such as aluminum, cobalt, copper, and nickel [11,12,13,14], and more than 500 siderophores with strong strain specificity having been identified in various bacterial species [15].

The bacterial genus *Pseudomonas* comprises highly diverse and dominant species that range from plant, animal, and human pathogens to soil inhabitants. A number of *Pseudomonas* species, including the human opportunistic pathogen *Pseudomonas aeruginosa* and the plant pathogen *Pseudomonas syringae*, can produce a yellow-green fluorescent siderophore called pyoverdine (PVD; for the chemical structure of PVD, see Figure S1) [16]. PVD was first discovered in 1982, and more than 60 types of PVD produced by various *Pseudomonas* strains have been characterized [17,18]. The structure of these PVDs are generally composed of three parts: a conserved dihydroxyquinine chromophore synthesized by the NRPS pathways [12,19], with characteristic fluorescence observed at an excitation wavelength of 400 nm and an emission of 450 nm [10]; an acyl side chain (dicarboxylic acid or its monoamide) that binds to the amino groups of the chromophore, primarily succinic acid, malic acid, or an aminated derivative thereof; and a peptide chain that binds to the carboxyl group of the chromophore exhibiting highly variable length and composition and determining the OM receptor-binding specificity of PVDs [20]. The binding sites of PVD with ferric iron are typically provided by one catecholate and two hydroxamate groups, generating a high affinity for Fe^3+^ (*K_aff_* = ~10^24^ mol L^−1^) [21].

In addition to meeting the iron requirements of *Pseudomonas* strains, PVD is also essential for the infection and colonization of host tissues by strains that are pathogenic toward animals or plants. Furthermore, the ability of PVD-deficient *P. aeruginosa* mutants to cause infection is significantly reduced [22,23,24], and PVD has been demonstrated to be an important colonization factor in *P. syringae* that is required for the production of the toxin tabtoxin [25].

*P. syringae* is generally perceived to be an important plant pathogen that causes foliar necroses in host plants and a hypersensitive reaction in nonhosts, and it is an ice nucleation-active pathogen that causes frost injury to plants at subfreezing temperatures (as high as −2 to −5 °C) [26]. Interestingly, after genome sequencing and comparative genomic analysis of the wild-type *P. syringae* strain MB03, which has high ice-nucleating and infectious activities towards various crops, such as tomato, cucumber, and wheat [27], it was found that many of the virulence genes identified in this bacterium were identical to those present in the genome of *P. aeruginosa* [28,29], a bacterium with nematocidal activity towards the nematode *Caenorhabditis elegans* that uses a variety of virulence proteins against this worm [30,31]. In fact, we previously confirmed that the whole-cell pathogenic activity of MB03 against *C. elegans* [32] and the virulence factor YqfO03, which was expressed and purified from MB03, also exhibited nematocidal activity against *C. elegans* and the plant parasitic nematode *Meloidogyne incognita* [33]. Various other virulence determinants, such as two component systems (*phoQ/phoP* and *phoR/phoB*), flagellar proteins, and some proteases (*pepP*, *clpA*, and *clpS*) have been proposed to promote the pathogenicity of *P. syringae* against *C. elegans* [32]. However, despite intense study, the major virulence factors and their mechanisms of action in this bacterium have remained unelucidated and the roles of many virulence determinants, including siderophores, still needed to be evaluated. 

The current study investigated the role of PVD using a *P. syringae*–*C. elegans* liquid-based pathogenicity model. Following the identification of the gene cluster encoding proteins involved in PVD biosynthesis in the *P. syringae* MB03 genome using Anti-SMASH (Antibiotics and secondary metabolites analysis shell) and the prediction of its peptide chain composition using the online bioinformatics tool NRPS predictor, two NPRS genes (*pvdD* and *pvdJ*) involved in PVD biosynthesis were disrupted using a Rec/TE recombination system to generate the mutant strains MB03*∆pvdD* and MB03*∆pvdJ*, respectively. The effect of these gene disruptions on PVD production in MB03 was investigated using liquid chromatography-mass spectrometry (LC-MS) and spectrofluorimetric analyses, and the virulence of the mutant strains against *C. elegans* was examined via liquid killing (LK) bioassays. The role of iron and PVD in *C. elegans* killing was further investigated in the mutant- and MB03-mediated LK bioassays, and possible killing mechanisms were also discussed.

## 2. Results

### 2.1. P. syringae MB03 has the Genetic Potential to Produce PVD

**Figure 1:**
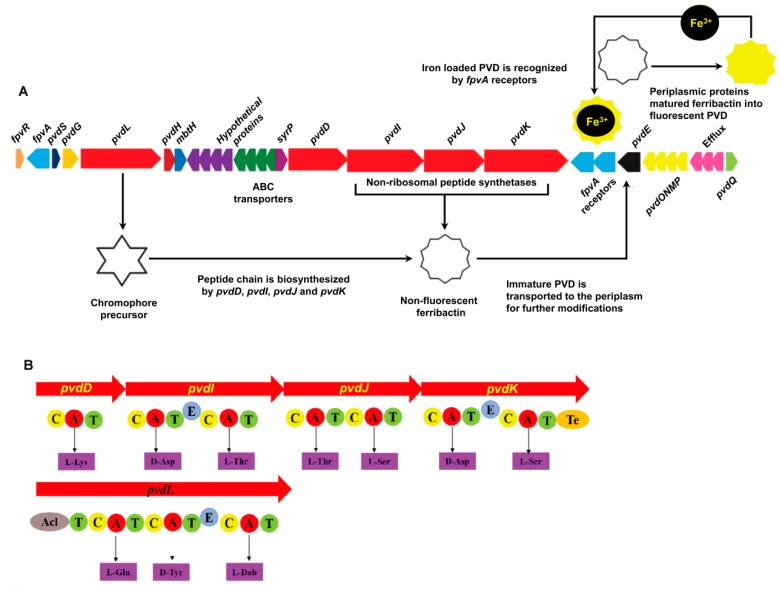
Genes involved in the biosynthesis of PVD in *P. syringae* MB03. (**A**) PVD genetic locus in the *P. syringae* MB03 genome: The red-colored genes encode non-ribosomal peptide synthetase (NRPS) enzymes (*pvdL/D/I/J/K*), which are involved in the biosynthesis of PVD. The dark blue colored-gene (*pvdS*) encodes an (extracytoplasmic function) ECF σ factor involved in the regulation of PVD biosynthetic and receptor genes, while the orange-colored gene (*fpvR*) encodes the anti-σ factor. Triplicate *fpvA* receptor genes are colored light blue; they are involved in the import of iron-loaded PVD into the cell. The immature PVD is transported into periplasm via the *pvdE* ABC (ATP-binding cassette) transporter, where a number of periplasmic proteins modify it to produce the mature PVD. *SyrP*, indicated in the purple color is an aspartate hydroxylase predicted to be involved in the β-hydroxylation of aspartate residues present in the PVD peptide chain. (**B**) Non-ribosomal peptide synthetases encoded in the MB03 PVD genetic cluster: Each enzyme consists of different domains, such as condensation (C), adenylation (A), thiolation (T), epimerization (E), and thioesterase domains (Te). Ten adenylation domains in the putative NRPS and the resulting peptides of each adenylation domain were predicted using NRPS predictor.

The predicted PVD genes were observed in one large cluster in the *P. syringae* MB03 genome (Figure 1) in contrast to other fluorescent pseudomonads, where they are separated into two or three clusters [19]. The structural organization of the PVD genes in *P. syringae* MB03 was highly similar to that observed in other *P. syringae* strains, such as SM and DC3000, showing 97% and 100% sequence similarity, respectively (results from anti-SMASH). Moreover, as almost all of the PVD genes of *P. syringae* MB03 could be identified in the *P. aeruginosa* PAO1 genome, we used the PAO1 genome as a reference model for PVD gene annotation in MB03 by alignment analysis, with the genes being considered orthologs when they had the same clusters of orthologous groups of proteins. The results identified 5 PVD genes in PAO1 that were not present in MB03 (i.e., two regulatory factor-encoding genes, namely, *pvdX* and *pvdY*; two biosynthetic enzyme-encoding genes, *pvdA* and *pvdF*; and a sigma factor-encoding gene, *fpvI*), while 29 other PVD genes were present in both strains (Table S1). The gene *VT47_09165* probably encodes an enzyme involved in the synthesis of the PVD chromophore, as it shares 72.3% predicted amino acid (AA) identity with the chromophore NRPS of PAO1. Moreover, this gene was observed to be significantly identical to those present in other fluorescent pseudomonads, as this gene is involved synthesizing the most conserved region of PVD. It is worth mentioning that *P. syringae* MB03 harbors three orthologs of the PAO1 ferripyoverdine receptor gene *fpvA*. The predicted products of these genes share 51.87% AA identity with one another in MB03 and 35.71% (*VT47_09095*), 34.59%, (*VT47_09245*), and 31.04% (*VT47_09250*) sequence similarities with *fpvA* from PAO1. A gene encoding a putative acylase (*VT47_09295*) was identified in the PVD genetic cluster of MB03. A three-gene cluster (*VT47_09290*, *VT47_09285*, and *VT47_09280*) has been shown to encode a membrane fusion protein, an ATP-binding permease, and an outer membrane protein, respectively, and to serve as an efflux pump that exports PVD out of the cell. Four adjacent NRPS genes, *pvdD, pvdI, pvdJ,* and *pvdK* (*VT47_09225*, *VT47_09230*, *VT47_09235*, and *VT47_09240*, respectively), were identified that are responsible for the production of peptide chain. NRPSs are large multimodular enzymes [34] in which each module is accountable for the integration of one AA into the peptide chain. The number and order of modules typically dictates the number and order of AAs in the peptide chain [19]. In each module, there are different domains, i.e., condensation (C), adenylation (A), thiolation (T), and epimerization (E) domains. As the name indicates, the adenylation domain is responsible for identification and activation of a specific AA via its acyl adenylate activity by reacting with ATP in each module. Ten adenylation domains were detected by bioinformatics analysis of MB03-PVD biosynthetic genes. The specificity of the A domain for the *P. syringae* MB03 PVD side chain was predicted using an online bioinformatics tool described in the Materials and Methods section. The first AA of the peptide chain is L-Lys (signature residues: DGEDHGTV). The second AA is predicted to be D-Asp (signature residues: DLTKIGHV, due to presence of a downstream epimerization domain it is in D-configuration). The third and fourth AAs are predicted to be L-Thr (the signature residues of these AAs are the same DFWNIGMV). The fifth to seventh AAs are L-Ser, D-Asp, and L-Ser (the signature residues are DVMHVSLI, DLTKIGHV, and DVWHVSLI, respectively). All *P. syringae* pathovars produce the same type of PVD, including MB03, showing a similar pattern with other *P. syringae* and *P. aeruginosa* strains.

### 2.2. PVD is Only Produced by P. syringae MB03 under Iron-limiting Conditions

**Figure 2:**
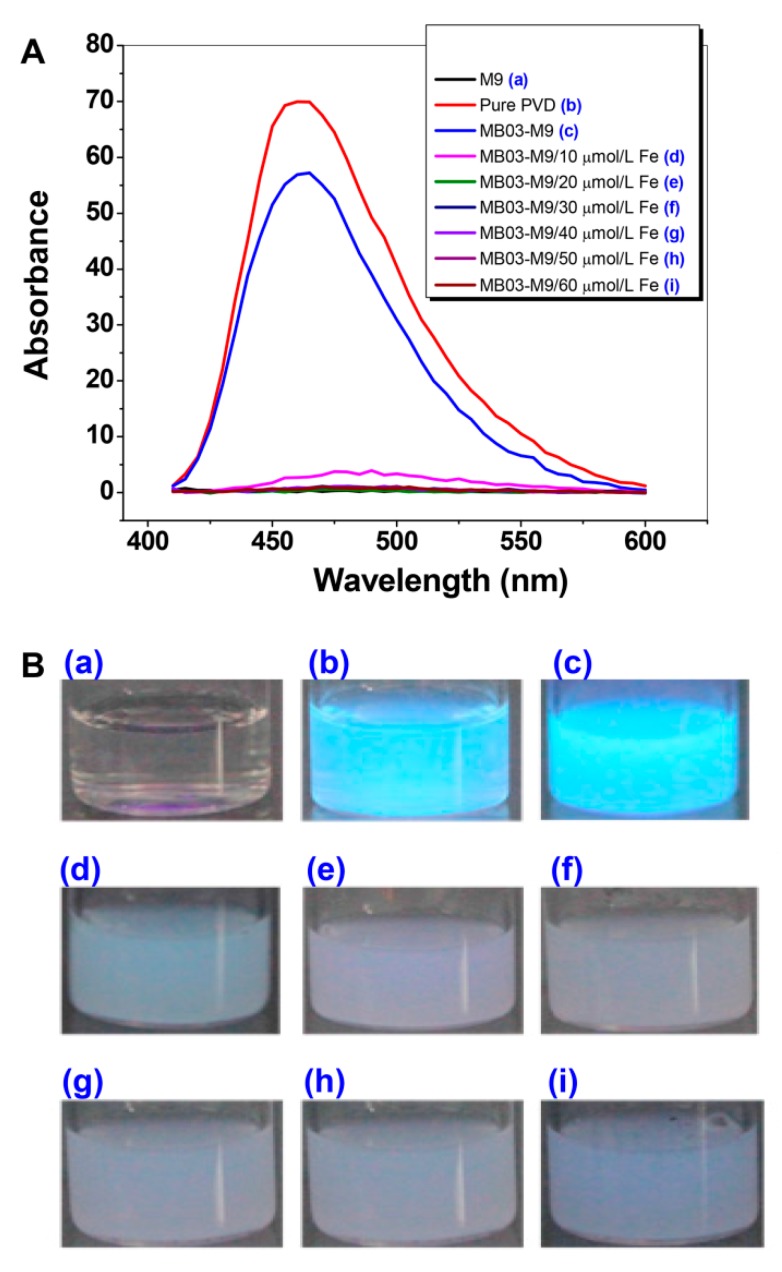
Production of PVD by *P. syringae* MB03 is negatively controlled by iron. (**A**) Wavelength (400 to 600 nm)-scanning curves of the supernatants of *P. syringae* MB03 cultures supplemented with different concentrations of exogenous iron. (**B**) Fluorescent supernatants of *P. syringae* MB03 culture suspensions. (**a**) M9 medium; (**b**) pure PVD; and (**c**)−(**i**), the supernatants from the cultures supplemented with exogenous iron at 0, 10, 20, 30, 40, 50, and 60 µmol L^-1^, respectively.

To verify the function of iron in the production of PVD, *P. syringae* MB03 cells were grown with or without exogenous iron supplementation at concentrations ranging from 10 to 60 µmol L^−1^. The diluted supernatants produced absorbed light from 450 nm to approximately 600 nm, with the highest peak at 460 nm. As shown in Figure 2, PVD was produced under conditions of no or low levels (10 µmol L^−1^) of iron supplementation, indicating that PVD is only produced by MB03 under iron-limiting conditions. This result is consistent with that of a previous investigation by Yin et al. [35]. Based on these results, we considered that 20 µmol L^-1^ of iron would be a crucial dose for PVD production in MB03.

### 2.3. Mutants MB03∆pvdD and MB03∆pvdJ Fail to Produce PVD in MB03

**Figure 3:**
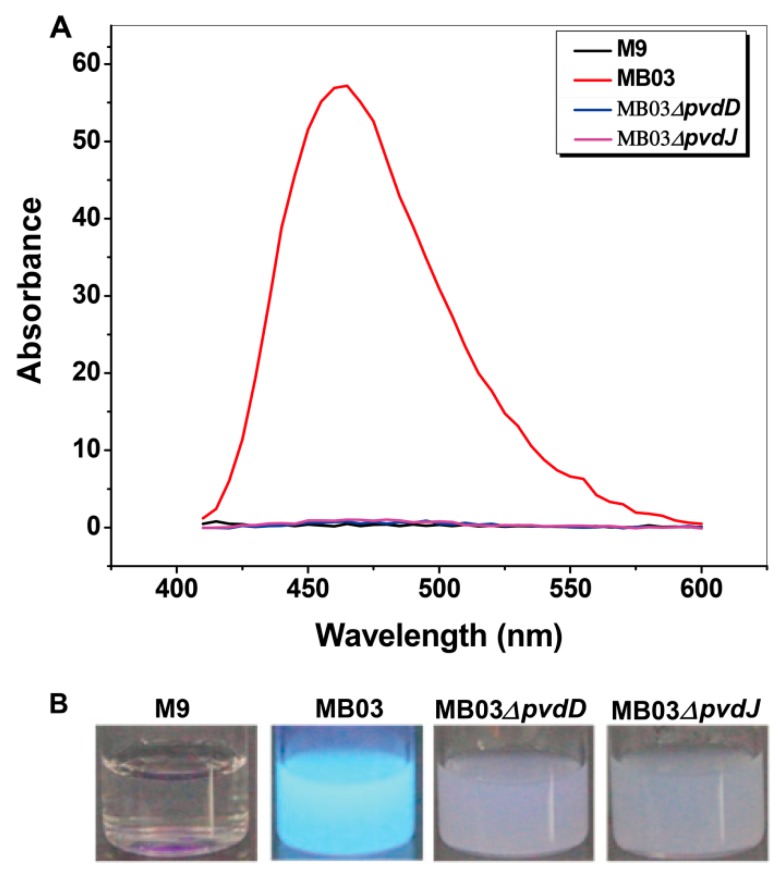
PVD production in *P. syringae* MB03 and the mutants MB03*∆pvdD* and MB03*∆pvdJ*: (**A**) Wavelength-scanning curves of the supernatants from the *∆pvdD* and *∆pvdJ* mutant strain culture suspensions and (**B**) fluorescence of *P. syringae* MB03, MB03*∆pvdD*, and MB03*∆pvdJ* cell suspensions. M9 was used as the negative control.

**Figure 4:**
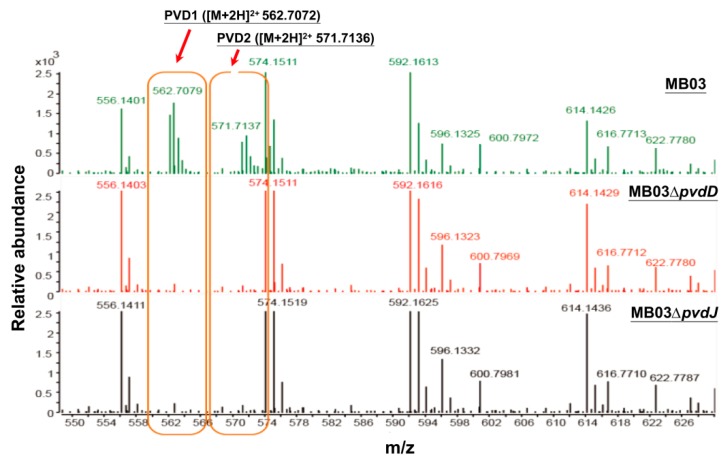
Liquid chromatography-mass spectrometry (LC-MS) analysis of cell free filtrates of MB03, MB03*∆pvdD*, and MB03*∆pvdJ*: MB03 and its derivatives were grown in M9 media for 48 h.

**Figure 5:**
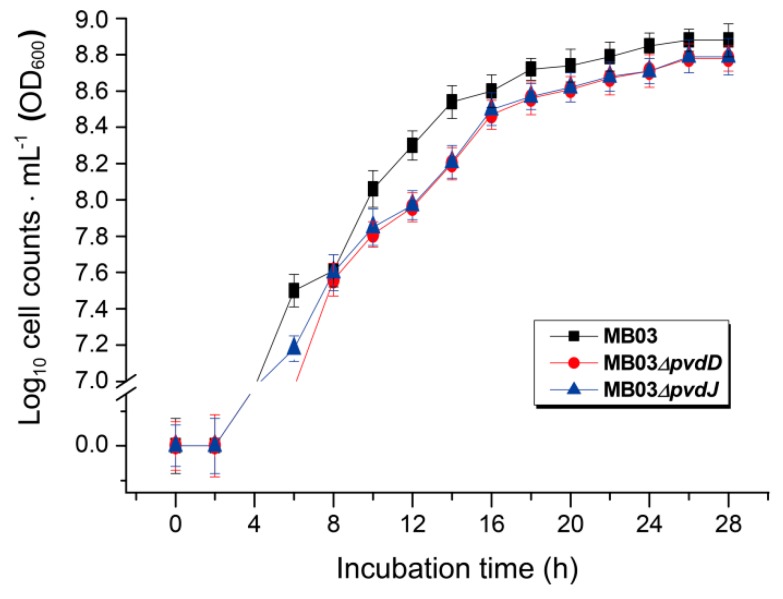
The growth curves of *P. syringae* MB03 and the mutant strains MB03*∆pvdD* and MB03*∆pvdJ*: MB03 and its derivatives were grown in M9 media for 28 h.

The NPRS genes *pvdD* and *pvdJ* encode non-ribosomal peptide synthetase proteins. To investigate the roles of *pvdD* and *pvdJ* in the biosynthesis of PVD in MB03, *pvdD*- and *pvdJ*-disrupted mutants were constructed using a Rec/TE recombination system (Figure S2). As PVD is a secreted non-ribosomal polypeptide, the cell-free filtrates from the wild-type strain MB03 and the mutants MB03*∆pvdD* and MB03*∆pvdJ* were analyzed using a fluorospectrophotometer and an LC-MS assay. The results showed that, compared to the substantial production of PVD in MB03, both the MB03*∆pvdD* and MB03*∆pvdJ* strains failed to produce PVD in the normalized spectrofluorometric assays of the cell-free filtrates (Figure 3A) and no visible fluorescence could be observed in both MB03*∆pvdD* and MB03*∆pvdJ* cell suspensions, whereas the MB03 suspension showed strong fluorescence (Figure 3B). Like most fluorescent pseudomonads, which produce several PVD isoforms (primarily due to differences in the dicarboxylic acid side chain) when grown in iron-deficient medium, MB03 was also able to produce two PVDs: 1123.412 and 1142.416 m/z. The LC-MS data showed that, compared to the two distinctive peaks observed for PVD1 and PVD2 in MB03, no corresponding peak could be detected for the MB03*∆pvdD* and MB03*∆pvdJ* mutants (Figure 4). These results suggest that both *pvdD* and *pvdJ* are required for the production of PVD in MB03. Interestingly, the growth of both the MB03*∆pvdD* and MB03*∆pvdJ* mutants appeared to be restricted, comparted to that of the parent strain due to iron deficiency (Figure 5), indicating that PVD is also required for the normal growth of the host cells.

### 2.4. PVD from P. syringae MB03 Promotes the Death of C. elegans

**Figure 6:**
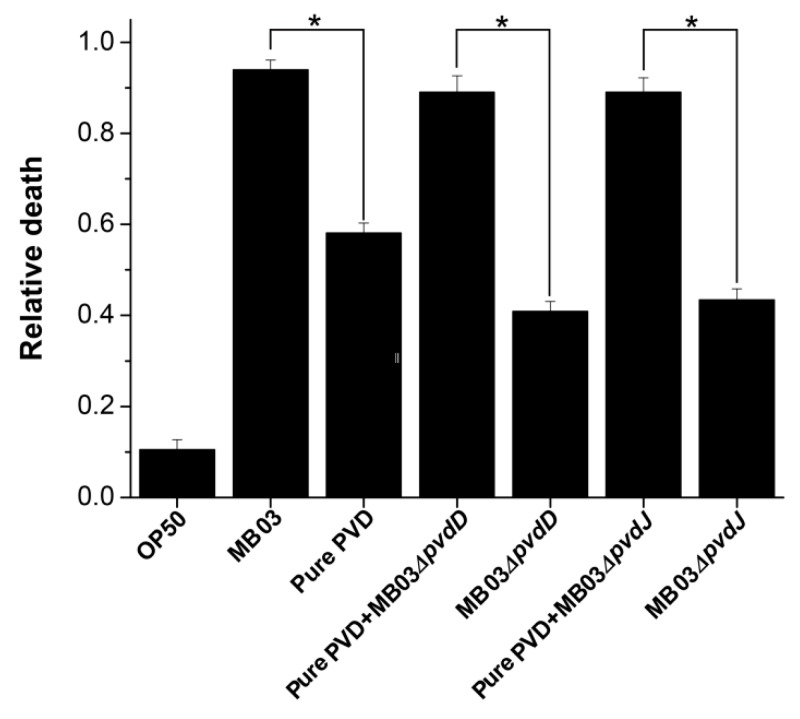
Nematocidal activity assay of *P. syringae* MB03 and the mutant strains MB03*∆pvdD* and MB03*∆pvdJ*, alone or with pure PVD: MB03 filtrate (MB03 grown in M9 which induce PVD production) showed noteworthy killing. The role of PVD in liquid killing (LK) assay was confirmed by attenuated virulence of both mutants (defective in PVD production). Pure PVD was added in the mutants to measure the relative death rate, which was comparable with the wild-type MB03. Mortality of worms in each well was determined in 4 days. Asterisks indicate a significant difference between the selected two groups in a two tailed t test (*p* value < 0.05). Three biological replicates were performed for each experiment. Error bars represent the standard deviations from the means of three independent experiments.

To determine whether PVD is associated with the nematocidal activity of *P. syringae* MB03 against free-living nematode *C. elegans* [32], we performed an LK bioassay to evaluate *C. elegans* mortality using cell-free filtrates of the wild-type MB03 strain and both mutants (MB03*∆pvdD* and MB03*∆pvdJ)* strains, which are unable to produce PVD under iron-limiting conditions. In addition, the commercially available pure PVD was assayed at a normalized dose that was comparable with that of the PVD produced by MB03 after 48 h. As shown in Figure 6, the filtrate from MB03 exhibited the highest nematocidal activity, while the pure PVD also showed substantial nematocidal activity. Not unexpectedly, the nematocidal activity of the filtrates from both the MB03*∆pvdD* and MB03*∆pvdJ* mutants was remarkably decreased compared to that of MB03. However, the nematocidal activity of both mutants was restored to a level that was similar to that of the parent strain when an almost equivalent amount of pure PVD was added to compensate for the lack of PVD in the mutant filtrates. Thus, these results indicate that the PVD from *P. syringae* MB03 is responsible for the observed mortality of the worms and functions as a nematocidal virulence factor against *C. elegans*. Moreover, the observation that the nematocidal activity of the filtrates from both mutants was not completely lost indicated the presence of additional virulence factors in addition to PVD.

### 2.5. Role of Iron in the MB03–C. elegans Interaction

**Figure 7:**
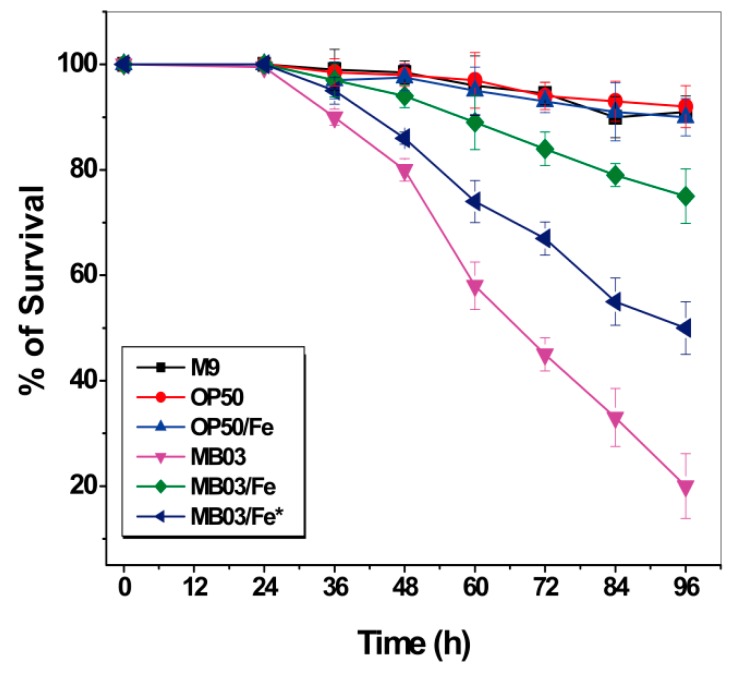
Effect of additional accessible iron on the nematocidal activity of *P. syringae* MB03 against *C. elegans*: Survival of worms when exposed to M9, OP50 (OP50 was grown in M9), OP50/Fe (OP50 was grown in M9 with 100 µmol L^−1^ iron), MB03 (MB03 was grown in M9), MB03/Fe (MB03 was grown in M9 with 100 µmol L^−1^ iron), and MB03/Fe* (the filtrate generated from 48 h MB03 culture in M9 and then incubated with 100 µmol L^−1^ iron overnight) filtrates in LK assay. M9 and OP50 was used as control. MB03 filtrate showed significant killing that was attenuated by addition of exogenous iron while growth of MB03 in M9 or by overnight incubation of MB03 filtrate was attenuated with 100 µmol L^−1^ iron. The mortality of worms in each well was determined in every 12 h. Three biological replicates were used, and 40–50 worms were added per replicate. Error bars represent the standard deviations from the means of three independent experiments.

Iron starvation conditions cause a robust tug-of-war between the host and pathogen for available iron. We speculated that the nematocidal activity of *P. syringae* MB03 toward *C. elegans* worms may be due to their ability to compete for environmentally available iron resources. The ability of MB03 cells to produce PVD confers them an advantage over worms with respect to their ability to capture more iron, resulting in a hypoxic response that indirectly kills the worms. Therefore, we investigated the effect of additional accessible iron on the nematocidal activity of *P. syringae* MB03 against *C. elegans*. To this end, exogenous iron was supplemented at 100 µmol L^-1^ to either the initial culture medium of MB03 to ensure an adequate supply of iron throughout the growth process or to the filtrate obtained from a 48-h cell suspension without additional iron during the cultivation. As shown in Figure 7, while both *E. coli* OP50 cells alone and the OP50 cells/Fe (the cells were cultured with 100 µmol L^−1^ iron supplementation) exhibited negative nematocidal activity against *C. elegans* (legend: OP50 and OP50/Fe), MB03 cells alone exhibited normal high activity against the worms (legend: MB03). However, the addition of exogenous iron significantly attenuated the nematocidal activity of the cells (legend: MB03/Fe) compared to that observed under iron-limiting conditions (legend: MB03). These results suggest that iron could be a crucial virulence determinant during the course of *P. syringae* MB03 infection of *C. elegans* worms. When sufficient iron was available, MB03 and *C. elegans* could coexist, whereas the infection of the worms by MB03 was obvious under iron-deficient conditions. Interestingly, iron exerted a counteractive effect on MB03 cells cultured for 48 h that were believed to produce adequate PVD, and then, this filtrate incubated with 100 µmol L^-1^iron overnight (legend: MB03/Fe*). PVD was previously reported to serve as a signaling molecule by inducing the production of a number of virulence factors, such as fpvA, endoprotease PrpL, and exotoxin A in *P. aeruginosa* [36,37] and tabtoxin, extracellular polysaccharide, and acyl homoserine lactones in *P. syringae* [25] (Figure S3). Therefore, we hypothesized that the overnight incubation of MB03 filtrate with exogenous iron may decrease the production of these pathogenic factors in MB03.

## 3. Discussion

We have recently reported the pathogenicity of a notorious plant-pathogenic *P. syringae* strain against the animal model organism *C. elegans* [32], but the virulence determinants and their mode of action remain unelucidated. The goal of the current study was to investigate the role of a siderophore called pyoverdine and ferric iron in the pathogenicity of *P. syringae* MB03 against *C. elegans* worms. Although the hypoxic response induced by *P. aeruginosa*-produced PVD was previously shown to promote the death of *C. elegans* worms, the activity of PVD from *P. syringae* against *C. elegans* has not been reported to date. Using a liquid-based pathogenicity model, we investigated the association between PVD and iron with respect to the pathogenicity of *P. syringae* toward *C. elegans*. Collectively, our results clearly showed that PVD produced by *P. syringae* MB03 was enough to kill *C. elegans* worms. In addition, whereas MB03 induced PVD production to promote *C. elegans* killing under iron-limited conditions, sufficient iron availability compromised the virulence of MB03.

The PVD gene cluster in *P. syringae* MB03 is structurally different from that of *P. aeruginosa* PAO1 due to the lack of 5 genes, i.e., *pvdX*, *pvdY*, *pvdA*, *pvdF*, and *fpvI* (Table S1), consistent with the findings of a previous investigation [38]. In *P. aeruginosa, fpvI* is necessary for the expression of *fpvA* (TonB-dependent ferri-PVD receptor), although it is not directly involved in the biosynthesis of PVD [39]. Previous investigations have shown that *fpvI* is not present in the genomes of certain *P. syringae* strains; instead, another gene, namely, *pvdS*, was observed to be involved in the transcription of *fpvA* [4,19]. The genes *pvdA* and *pvdF* are required for the conversion of ornithine residues in the *P. aeruginosa* PVD peptide chain into hydroxyornithine and then into formyl hydroxyornithine, respectively [40], and a mutation in *pvdY* abrogated PVD production in *P. aeruginosa* [41]. However, the exact function of this gene, together with *pvdX*, remains unknown. Although the *P. syringae* PVD peptide chain does not contain ornithine residues, PVD typically harbor three iron-binding ligands, with one situated in chromophore part and the other two being located in the peptide chain [42]. Moreover, PVD-mediated iron uptake in *P. syringae* was reported to be highly similar to that in *P. aeruginosa* [25]. Thus, these previous findings suggest that, although PVD from *P. syringae* MB03 and PAO1 are structurally different, their iron scavenging properties could be similar. 

Siderophore biosynthesis and iron acquisition are essential for *P. aeruginosa* virulence in both plants and animals [43,44]. The nematocidal activity of the siderophore PVD has been reported in *P. aeruginosa*, revealing that PVD alone is sufficient to kill *C. elegans* [30]. In *P. syringae*, we previously showed that the genes required for PVD synthesis, such as *pvdE* and *pvdJ*, *were significantly upregulated during the interaction of P. syringae MB03 and C. elegans worms* under pathogen-favorable conditions [32], raising the possibility that PVD is involved during the infection of *C. elegans* by *P. syringae*. It is noteworthy that the gene disruption-mediated loss of PVD production remarkably attenuated *P. aeruginosa* virulence in the LK assay but not in agar-based assays [30]. Therefore, we performed LK bioassays to investigate the role of PVD in the ability of *P. syringae* to kill *C. elegans* worms by deleting the PVD biosynthetic genes *pvdD* and *pvdJ*, and the pathogenicity of the mutants was compared with that of the wild-type strain. Consistent with previous investigations [25,30], the results showed complete abolition of PVD production by these mutants, and their virulence toward *C. elegans* was also compromised when compared to that of wild-type MB03. There is even a difference in growth rate of MB03 and mutants, while the concentration of extracellular components except PVD is the same as that in Figure 4. In another study, the growth rate of the *pvdJ* mutant was similar to that of the wild-type strain, but this mutant was still unable to produce PVD [25], which identified that the inability of mutant to produce PVD is not due to defect in growth rate. For this, we give the reason that the defect in growth rate was due to iron deficiency. To exclude the possibility that the loss of PVD production was caused by secondary mutation or polar effects, the commercially available pure PVD was also used in the current study to verify the role of PVD by using it to complement the mutants, which resulted in comparable activities with that of the wild-type strain. In another investigation, loss of *pvdD* and *pvdJ* caused complete abolition of PVD biosynthesis and complementation of *pvdJ* mutant resulted in the retention of PVD production [38]. However, only limited killing activity was observed by either the *pvdD*- or *pvdJ*-disrupted mutants. This result suggests that PVD not only disrupts the iron homeostasis of *C. elegans*, which results in the death of *C. elegans* worms, but also may regulate and/or arouse other virulence factors, such as exotoxins (i.e., tabtoxin and ExoA), endoproteases (PrpL), and exopolysaccharide production during pathogenesis [25,37]. Collectively, our results suggest that PVD is the crucial factor in the cell filtrates causing the death of *C. elegans*. In mammals, one ferritin protein has been proven to act as a storage protein and to serve as buffer during iron deficiency and overloaded conditions [45]. In *C. elegans*, there are two genes, namely, *ftn-1* and *ftn-2*, encoding homologs of this protein [46]. It is therefore of interest to investigate whether the proteins encoded by these genes in *C. elegans* have some function toward PVD.

Almost all organisms require iron for their growth and survival [47], and the ability to acquire iron is considered an integral factor in virulence [48]. Iron limitation initiates an ongoing struggle between the host and pathogen. Some pathogenic bacteria of plants and animals use common features to cause infection, including virulence-associated mechanisms for iron obtaining, i.e., siderophore-mediated iron sequestration [25,30], conserved systems for deploying virulence proteins [49], and centralized pathogenic strategies [50]. According to previous investigations, PVD biosynthesis is negatively regulated by the presence of intracellular iron [51]. Taken together, sufficient iron availability was hypothesized to potentially decrease PVD production and to attenuate the virulence of *P. syringae* against *C. elegans* in a liquid-based infection model. To investigate the role of iron in the *P. syringae–C. elegans* interaction, different filtrates produced from *P. syringae* MB03 with or without iron supplementation were used in the assays, and the pathogenicity of MB03 grown in different conditions was compared. Unsurprisingly, the filtrate from MB03 grown in M9 (a condition that induces PVD production) showed significant killing that was attenuated by the addition of exogenous iron (a condition that limits PVD production) during the growth of MB03 in M9 medium. An incubation of the filtrate produced from the growth of MB03 in M9 medium with iron also attenuated the killing of worms. Moreover, we also observed that iron replete conditions retarded PVD production and substantially decreased *C. elegans* death. A potential reason for these observations is that, because host iron is important for numerous biological processes, PVD may directly function as a worm-killing toxin via iron sequestration [30].

## 4. Materials and Methods 

### 4.1. Bacterial and C. elegans Strains, Plasmids, Media, Chemicals, and Culture Conditions

The bacterial strains and plasmids used in this study are listed in Table 1. Briefly, the wild-type *P. syringae* strain MB03 [27] was investigated for its pathogenicity against the model organism *C. elegans*. The *pvdD*- and *pvdJ*-disrupted mutant strains of MB03 (namely, MB03*∆pvdD* and MB03*∆pvdJ*, respectively) were constructed to examine the function of these *pvd* genes with respect to the production of PVD and in MB03 pathogenicity against *C. elegans*. The wild-type *C. elegans* strain N2 was provided by the *Caenorhabditis* Genetics Center (CGC) (College of Biological Sciences, University of Minnesota, MN55108, USA) and was maintained at 20 °C on nematode growth medium (NGM) agar plates using *Escherichia coli* OP50 as food. The synchronized L4 stage worms of the wild-type *C. elegans* strain N2 were utilized for the LK assay. 

*P. syringae* and *E. coli* strains were cultured in lysogeny broth (LB) [52] at 28 °C and 37 °C, respectively, for routine growth. To culture recombinant strains, the antibiotics ampicillin, gentamycin, or kanamycin were used as need at final concentrations of 100, 10, and 50 µg mL^−^^1^, respectively. For PVD assays, FeCl_3_ was added to M9 minimal medium (comprising 200 mL L^−^^1^ M9 salts (33.5 g L^−^^1^ Na_2_HPO_4_, 15 g L^−^^1^ KH_2_PO_4_, 2.5 g L^−^^1^ NaCl, and 5 g L^−^^1^ NH_4_Cl), 2 mL L^−^^1^ 1 mol L^−^^1^ MgSO_4_, 20 mL L^−^^1^ 20% glucose, and 0.1 mL L^−^^1^ 1 mol L^−^^1^ CaCl_2_) at final concentrations of 10, 20, 30, 40, 50, and 60 µmol L^−^^1^. A PVD standard (≥99% purity) was purchased from Sigma-Aldrich Co. Chromatographic-grade methanol and acetonitrile (≥99.8 purity) were purchased from MREDA (China Branch, Beijing 100096, China). All other chemicals and reagents used were of analytical grade.

### 4.2. Direct de novo Genome Mining of MB03 to Identify PVD-encoding Biosynthetic Genes

The PVD genetic clusters of *P. syringae* MB03 were predicted by analyzing the whole genome sequence of MB03 (DDBJ/EMBL/GenBank under accession LAGV00000000) with the antibiotics and secondary metabolite analysis shell (Anti-SMASH) pipeline [54]. Subsequently, amino acid (AA) sequences of well-identified PVD genes of *P. aeruginosa* PAO1 were aligned against the *P. syringae* MB03 genome by conducting BLASTp search of the GenBank AA sequence database at the National Center for Biotechnology Information (NCBI) server (http://blast.ncbi.nlm.nih.gov/Blast.cgi). The peptide chain composition and signature residues of *P. syringae* MB03 PVD were predicted by the AA sequence of genes encoding the corresponding non-ribosomal peptide synthetases utilizing online bioinformatics tool “NRPS predictor” (http://nrps.informatik.uni.tubeingen.de/) [55].

### 4.3. PVD Determination Assay

The PVD concentration was quantified using a previously described protocol [56] with few modifications. After growing *P. syringae* MB03 overnight in LB, the cell suspension was adjusted to an OD_600_ of 0.35 with sterile double-distilled water (ddH_2_O), inoculated into 5 mL M9 broth, and then incubated with agitation for 48 h at 28 °C. Following the incubation, the medium changed to a yellowish-green color, indicating the production of PVD. The cells were then pelleted by centrifugation at 10,000× *g* for 15 min at 4 °C. Subsequently, the supernatants were passed through a 0.22-µm filter membrane and then diluted 1:10 in 50 mM Tris⋅HCl. The quantity of PVD in the supernatants was calculated via fluorescence emission spectroscopy, with excitation and emission performed at 400 and 460 nm, respectively. M9 medium was used as a negative control, and a PVD standard was used as a positive control. All the experiments were performed three times.

### 4.4. Disruption of PVD Biosynthetic Genes using the RecTE Recombination System

The MB03*∆pvdD* and MB03*∆pvdJ* mutant strains were constructed via the Rec/TE recombination system (Figure S2) following previously described procedures [53,57]. Briefly, the plasmid pUCP24/recTE was initially transformed into *P. syringae* MB03 by electroporation using a previously described protocol [58]. The encoded RecE protein is an exonuclease that converts dsDNA to ssDNA [53,59], while RecT (also called annealing protein) binds to the newly formed ssDNA to protect it from degradation and promotes recombination at the homologous target site [53,59]. To construct the “upstream*-kan^r^-*downstream” recombinant gene fragment for the homologous double exchange of *pvdD* or *pvdJ* gene fragments in the *P. syringae* MB03 genome (Figure S2), separate PCRs were performed using the standard SOE (splicing by overlap extension) method [52]. Briefly, for *pvdD* disruption, 639-bp upstream and downstream fragments were amplified from the MB03 genome using the primer pairs *pvdD*up-F/*pvdD*up-R and *pvdD*dn-F/*pvdD*dn-R (Table 2), respectively, and a *Kan^r^* cassette was amplified from the plasmid pKD4 with the primer pair *pvdJ*neo-F and *pvdJ*neo-R (Table 2) to generate an integrated 2556-bp fragment using the PCR-SOE method. For *pvdJ* disruption, 665- and 678-bp upstream and downstream fragments were amplified from the MB03 genome using the primer pairs *pvdJ*up-F/*pvdJ*up-R and *pvdJ*dn-F/*pvdJ*dn-R (Table 2), respectively, to generate a 2520-bp triple fragment with the amplified *Kan^r^* cassette described above. To screen and identify the MB03*∆pvdD* and MB03*∆pvdJ* mutant strains, Kan-resistant colonies were selected from the plates. Subsequently, genomic DNA was obtained from these strains and then used as template for PCR amplification of the fused gene fragment “*frt-neo-frt^r^*” using the primer pairs *pvdD*seq-F/*pvdD*seq-R and *pvdJ*seq-F/*pvdJ*seq-R) for MB03∆*pvdD* and MB03∆*pvdJ*, respectively. 

### 4.5. LC-MS Analysis of Cell-Free Filtrates from MB03 and its Derivatives

LC-MS analysis was performed according to a published protocol [60] with some modifications. To ensure the sufficient production of PVD, MB03 (or its derivatives) was cultured for 48 h to a saturated suspension in M9 medium lacking iron. Subsequently, the cells were pelleted by centrifugation at 10,000× *g* for 15 min at 4 °C, and the supernatant was further filtered using a 0.2-µm pore-sized filter membrane. LC-MS analysis was performed using a Q-TOF (Quadrupole time of flight) LC/MS with an electrospray ionization ion source (4000 V capillary voltage, 30-V sampling cone voltage, 250 °C, nitrogen gas flow of 11 L min^-1^). Separation was performed on an Agilent Zorbax Eclipse Plus C18 narrow bore RR column (2.1 × 150 mm, 3.5 microns) using the following linear gradient of solvent A (double-distilled water with 0.1% (*v*/*v*) formic acid) and solvent B (acetonitrile with 0.1% (*v*/*v*) formic acid). The gradient started with 5–40% solution A for 0–10 min, then changed linearly to 40–5% solution A from 10–11 min, and finally culminated with 5% solution A at a flow rate of 0.3 mL min^-1^.

### 4.6. Efficacy of LK Assay

A 96-well LK bioassay system was used to evaluate the nematocidal activity of PVD produced by MB03 against the model organism *C. elegans* following a previously described method [30] with few modifications. Briefly, *E. coli* OP50, MB03, and/or its derivatives were cultured overnight in L; diluted to an OD_600_ value of 0.35; inoculated into 5 mL M9 broth with or without supplemented iron; and then incubated at 28 °C for an additional 48 h. The bacterial cells were then pelleted by centrifugation at 10,000× *g* for 15 min at 4 °C, and then, the supernatants were filtered through a 0.22-µm filter membrane. The cell-free filtrates were then used for bioassay experiments. *C. elegans* N2 worms were synchronized via the sodium hypochlorite isolation of eggs from gravid adult worms followed by the hatching of worms in M9 buffer. L1 larvae were then transferred to NGM plates seeded with *E. coli* OP50 and incubated at 22 °C for 44 h. The L4 stage worms were then rinsed from the plates and washed with M9 buffer. For the assay, each well contained 150 µL of cell-free filtrate, 7 µL of 8 mmol L^−1^ 5-fluorodeoxyuridine (FUdR; 0.2 mmol L^−1^ final concentration), 23 µL of M9, and 20 µL of L4 worms (40–50 worms). The commercially available pure PVD was added at a normalized dose that was comparable with that of the PVD produced by MB03 after 48 h. A dissection microscope was used to document the score of live, dead, or censored (animals that bagged, that burst, or that could not be located) animals*. C. elegans* worms were considered dead when they stopped moving and did not respond to a nudge with a platinum wire. Relative death rate was calculated using the following formula:

(1) $$Relative\;death\;rate=\frac{\mathrm{No}.\mathrm{of}\;\mathrm{dead}\;\mathrm{worms}}{\mathrm{No}.\;\mathrm{of}\;\mathrm{total}\;\mathrm{worms}}$$

### 4.7. Data Analysis

Data analysis was performed using Statistical Package for the Social Sciences (SPSS), version 13.0. Graphs were prepared using Origin 8 software (Origin Lab Corp., Northampton, MA, USA).

## 5. Conclusions

In summary, this is the first study to investigate the role of the iron-binding secreted toxin PVD in a *P. syringae*–*C. elegans* liquid-based pathogenicity model. The data showed clear evidence that iron plays a crucial role in the pathogenicity of MB03 against *C. elegans* and that PVD is the effective agent in the filtrates that promotes *C. elegans* death.

## Figures and Tables

**Table 1:** Bacterial strains and plasmids used in this study.

Bacterial Strains and Plasmids	Phenotypes	Sources or References
*P. syringae*		
MB03	A wild-type strain with nematocidal activity	[32]
MB03/RecTE	Transformed MB03 strain containing the plasmid pUCP24/*recTE* for the expression of the RecTE protein	This study
MB03*∆pvdD*	*pvdD*-disrupted mutant of MB03	This study
MB03*∆pvdJ*	*pvdJ*-disrupted mutant of MB03	This study
*E. coli* OP50	The food source used for *C. elegans* N2	Laboratory stock
Plasmids		
pUCP24/*recTE*	A recombinant plasmid harboring the *recTE* genes	[53]
pKD4	A plasmid harboring a kanamycin-resistance gene cassette	[53]

**Table 2:** Oligonucleotide primers used in this study.

Primers	Sequence
*pvdD* up-F	5′-CACGCAGTTGGTCGCCTA-3′
*pvdD* up-R	5′-GTTCCTATTCCGAAGTTCCCGGCAACAGGCTGAAATC-3′
*pvdD* neo-F	5′-GATTTCAGCCTGTTGCCGGGAACTTCGGAATAGGAAC-3′
*pvdD* neo-R	5′-TTCGCCATTTTTTCCCTGTCAGAAGAACTCGTCAAGAAG-3′
*pvdD* dn-F	5′-CTTCTTGACGAGTTCTTCTGACAGGGAAAAAATGGCGAA-3′
*pvdD* dn-R	5′-CGATGTGGTGCTGGGTCA-3′
*pvdD* seq-F	5′-TTCGCGGTTTCCGTATCGAGC-3′
*pvdD* seq-R	5′-GATCGGCAACGGTGCCAGTGT-3′
*pvdJ* up-F	5′-TGTCGGCGTACTTGGTGC-3′
*pvdJ* up-R	5′-GTTCCTATTCCGAAGTTCCTCTCCCTCACGGCAATCG-3′
*pvdJ* neo-F	5′-CGATTGCCGTGAGGGAGAGGAACTTCGGAATAGGAAC-3′
*pvdJ* neo-R	5′-CTCGAAGATCAGGCGCAGGAAGAACTCGTCAAGAAG-3′
*pvdJ* dn-F	5′-CTTCTTGACGAGTTCTTCTGACTGCGCCTGATCTTCGAG-3′
*pvdJ* dn-R	5′-GACCAGCCATCGGACACC-3′
*pvdJ* seq-F	5′-AAAATTCGCGGCTTCCGTATT-3′
*pvdJ* seq-R	5′-CGTAGTCGGCGTACTGAATCG-3′

## Supplementary Materials

Supplementary materials are available at https://www.mdpi.com/1422-0067/21/6/2198/s1.

## References

[1] Miethke M., Marahiel M.A. (2007). Siderophore-based iron acquisition and pathogen control. Microbiol. Mol. Biol. Rev..

[2] Ganz T., Nemeth E. (2015). Iron homeostasis in host defence and inflammation. Nat. Rev. Immunol..

[3] Andrews S.C., Robinson A.K., Rodriguez-Quinones F. (2003). Bacterial iron homeostasis. FEMS Microbiol. Rev..

[4] Chu B.C., Garcia-Herrero A., Johanson T.H., Krewulak K.D., Lau C.K., Peacock R.S., Slavinskaya Z., Vogel H.J. (2010). Siderophore uptake in bacteria and the battle for iron with the host; a bird’s eye view. Biometals.

[5] Wilson B.R., Bogdan A.R., Miyazawa M., Hashimoto K., Tsuji Y. (2016). Siderophores in iron metabolism: From mechanism to therapy potential. Trends Mol. Med..

[6] Hider R.C., Kong X. (2010). Chemistry and biology of siderophores. Nat. Prod. Rep..

[7] Koglin A., Walsh C.T. (2009). Structural insights into nonribosomal peptide enzymatic assembly lines. Nat. Prod. Rep..

[8] Wang H., Sivonen K., Fewer D.P. (2015). Genomic insights into the distribution, genetic diversity and evolution of polyketide synthases and nonribosomal peptide synthetases. Curr. Opin. Genet. Dev..

[9] Cezard C., Farvacques N., Sonnet P. (2015). Chemistry and biology of pyoverdines, *Pseudomonas* primary siderophores. Curr. Med. Chem..

[10] Folschweiller N., Gallay J., Vincent M., Abdallah M.A., Pattus F., Schalk I.J. (2002). The interaction between pyoverdin and its outer membrane receptor in *Pseudomonas aeruginosa* leads to different conformers: A time-resolved fluorescence study. Biochemistry.

[11] Braud A., Hannauer M., Mislin G.L., Schalk I.J. (2009). The *Pseudomonas aeruginosa* pyochelin-iron uptake pathway and its metal specificity. J. Bacteriol..

[12] Visca P., Imperi F., Lamont I.L. (2007). Pyoverdine siderophores: From biogenesis to biosignificance. Trends Microbiol..

[13] Baysse C., De Vos D., Naudet Y., Vandermonde A., Ochsner U., Meyer J.M., Budzikiewicz H., Schafer M., Fuchs R., Cornelis P. (2000). Vanadium interferes with siderophore-mediated iron uptake in *Pseudomonas aeruginosa*. Microbiology.

[14] Braud A., Hoegy F., Jezequel K., Lebeau T., Schalk I.J. (2009). New insights into the metal specificity of the *Pseudomonas aeruginosa* pyoverdine-iron uptake pathway. Environ. Microbiol..

[15] Cornelis P., Matthijs S. (2002). Diversity of siderophore-mediated iron uptake systems in fluorescent pseudomonads: Not only pyoverdines. Environ. Microbiol..

[16] Meyer J.M., Gruffaz C., Raharinosy V., Bezverbnaya I., Schafer M., Budzikiewicz H. (2008). Siderotyping of fluorescent *Pseudomonas*: Molecular mass determination by mass spectrometry as a powerful pyoverdine siderotyping method. Biometals.

[17] Budzikiewicz H. (2001). Siderophores of the human pathogenic fluorescent pseudomonads. Curr. Top. Med. Chem..

[18] Budzikiewicz H., Schafer M., Fernandez D.U., Matthijs S., Cornelis P. (2007). Characterization of the chromophores of pyoverdins and related siderophores by electrospray tandem mass spectrometry. Biometals.

[19] Ravel J., Cornelis P. (2003). Genomics of pyoverdine-mediated iron uptake in pseudomonads. Trends Microbiol..

[20] Meyer J.M. (2000). Pyoverdines: Pigments, siderophores and potential taxonomic markers of fluorescent *Pseudomonas* species. Arch. Microbiol..

[21] Budzikiewicz H. (2004). Siderophores of the Pseudomonadaceae sensu stricto (fluorescent and non-fluorescent *Pseudomonas* spp.). Fortschr. Chem. Org. Naturst..

[22] Kang D., Kirienko D.R., Webster P., Fisher A.L., Kirienko N.V. (2018). Pyoverdine, a siderophore from *Pseudomonas aeruginosa*, translocates into *C. elegans*, removes iron, and activates a distinct host response. Virulence.

[23] Handfield M., Lehoux D.E., Sanschagrin F., Mahan M.J., Woods D.E., Levesque R.C. (2000). In vivo-induced genes in *Pseudomonas aeruginosa*. Infect. Immun..

[24] Takase H., Nitanai H., Hoshino K., Otani T. (2000). Impact of siderophore production on *Pseudomonas aeruginosa* infections in immunosuppressed mice. Infect. Immun..

[25] Taguchi F., Suzuki T., Inagaki Y., Toyoda K., Shiraishi T., Ichinose Y. (2010). The siderophore pyoverdine of *Pseudomonas syringae* pv. tabaci 6605 is an intrinsic virulence factor in host tobacco infection. J. Bacteriol..

[26] Hirano S.S., Upper C.D. (2000). Bacteria in the leaf ecosystem with emphasis on *Pseudomonas syringae-*a pathogen, ice nucleus, and epiphyte. Microbiol. Mol. Biol. Rev..

[27] Li Q., Yan Q., Chen J., He Y., Wang J., Zhang H., Yu Z., Li L. (2012). Molecular characterization of an ice nucleation protein variant (InaQ) from *Pseudomonas syringae* and the analysis of its transmembrane transport activity in *Escherichia coli*. Int. J. Biol. Sci..

[28] Feinbaum R.L., Urbach J.M., Liberati N.T., Djonovic S., Adonizio A., Carvunis A.-R., Ausubel F.M. (2012). Genome-wide identification of *Pseudomonas aeruginosa* virulence-related genes using a *Caenorhabditis elegans* infection model. PLoS Pathogen..

[29] Dubern J.F., Cigana C., De Simone M., Lazenby J., Juhas M., Schwager S., Bianconi I., Döring G., Eberl L., Williams P. (2015). Integrated whole-genome screening for *Pseudomonas aeruginosa* virulence genes using multiple disease models reveals that pathogenicity is host specific. Environ. Microbiol..

[30] Kirienko N.V., Kirienko D.R., Larkins-Ford J., Wählby C., Ruvkun G., Ausubel F.M. (2013). *Pseudomonas aeruginosa* disrupts *Caenorhabditis elegans* iron homeostasis, causing a hypoxic response and death. Cell Host Microbe..

[31] Zaborin A., Romanowski K., Gerdes S., Holbrook C., Lepine F., Long J., Poroyko V., Diggle S.P., Wilke A., Righetti K. (2009). Red death in *Caenorhabditis elegans* caused by *Pseudomonas aeruginosa* PAO1. Proc. Natl. Acad. Sci. USA.

[32] Ali M., Sun Y., Xie L., Yu H., Bashir A., Li L. (2016). The pathogenicity of *Pseudomonas syringae* MB03 against *Caenorhabditis elegans* and the transcriptional response of nematicidal genes upon different nutritional conditions. Front. Microbiol..

[33] Manan A., Bazai Z., Fan J., Yu H., Li L. (2018). The Nif3-family protein YqfO03 from *Pseudomonas syringae* MB03 has multiple nematicidal activities against *Caenorhabditis elegans* and *Meloidogyne incognita*. Int. J. Mol. Sci..

[34] Lott J.S., Lee T.V. (2017). Revealing the inter-module interactions of multi-modular nonribosomal peptide synthetases. Structure.

[35] Yin K., Zhang W., Chen L. (2014). Pyoverdine secreted by *Pseudomonas aeruginosa* as a biological recognition element for the fluorescent detection of furazolidone. Biosens. Bioelectron..

[36] Beare P.A., For R.J., Martin L.W., Lamont I.L. (2003). Siderophore-mediated cell signalling in *Pseudomonas aeruginosa*: Divergent pathways regulate virulence factor production and siderophore receptor synthesis. Mol. Microbiol..

[37] Lamont I.L., Beare P.A., Ochsner U., Vasil A.I., Vasil M.L. (2002). Siderophore-mediated signaling regulates virulence factor production in *Pseudomonas aeruginosa*. Proc. Natl. Acad. Sci. USA.

[38] Owen J.G., Ackerley D.F. (2011). Characterization of pyoverdine and achromobactin in *Pseudomonas syringae* pv. phaseolicola 1448a. BMC Microbiol..

[39] Redly G.A., Poole K. (2003). Pyoverdine-mediated regulation of FpvA synthesis in *Pseudomonas aeruginosa*: Involvement of a probable extracytoplasmic-function sigma factor, FpvI. J. Bacteriol..

[40] Wilson M.J., McMorran B.J., Lamont I.L. (2001). Analysis of promoters recognized by PvdS, an extracytoplasmic-function sigma factor protein from *Pseudomonas aeruginosa*. J. Bacteriol..

[41] Lamont I.L., Martin L.W., Sims T., Scott A., Wallace M. (2006). Characterization of a gene encoding an acetylase required for pyoverdine synthesis in *Pseudomonas aeruginosa*. J. Bacteriol..

[42] Bultreys A., Gheysen I., Maraite H., de Hoffmann E. (2001). Characterization of fluorescent and nonfluorescent peptide siderophores produced by *Pseudomonas syringae* strains and their potential use in strain identification. Appl. Environ. Microbiol..

[43] Meyer J.M., Neely A., Stintzi A., Georges C., Holder I.A. (1996). Pyoverdin is essential for virulence of *Pseudomonas aeruginosa*. Infect. Immun..

[44] Nadal Jimenez P., Koch G., Papaioannou E., Wahjudi M., Krzeslak J., Coenye T., Cool R.H., Quax W.J. (2010). Role of PvdQ in *Pseudomonas aeruginosa* virulence under iron-limiting conditions. Microbiology.

[45] Truman-Rosentsvit M., Berenbaum D., Spektor L., Cohen L.A., Belizowsky-Moshe S., Lifshitz L., Ma J., Li W., Kesselman E., Abutbul-Ionita I. (2018). Ferritin is secreted via 2 distinct nonclassical vesicular pathways. Blood.

[46] Gourley B.L., Parker S.B., Jones B.J., Zumbrennen K.B., Leibold E.A. (2003). Cytosolic aconitase and ferritin are regulated by iron in *Caenorhabditis elegans*. J. Biol. Chem..

[47] Cherayil B.J. (2011). The role of iron in the immune response to bacterial infection. Immunol. Res..

[48] Lewis J.P. (2010). Metal uptake in host-pathogen interactions: Role of iron in *Porphyromonas gingivalis* interactions with host organisms. Periodontol. 2000.

[49] Van Gijsegem F., Genin S., Boucher C. (1993). Conservation of secretion pathways for pathogenicity determinants of plant and animal bacteria. Trends Microbiol..

[50] Collmer A., Bauer D.W. (1994). Erwinia chrysanthemi and *Pseudomonas syringae*: Plant pathogens trafficking in extracellular virulence proteins. Curr. Top. Microbiol. Immunol..

[51] Cornelis P., Matthijs S., Van Oeffelen L. (2009). Iron uptake regulation in *Pseudomonas aeruginosa*. Biometals.

[52] Sambrook J., Russell D.W. (2011). Molecular Cloning: A Laboratory Manual.

[53] Bao Z., Cartinhour S., Swingle B. (2012). Substrate and target sequence length influence RecTE(Psy) recombineering efficiency in *Pseudomonas syringae*. PLoS ONE.

[54] Blin K., Medema M.H., Kazempour D., Fischbach M.A., Breitling R., Takano E., Weber T. (2013). antiSMASH 2.0-a versatile platform for genome mining of secondary metabolite producers. Nucleic Acids Res.

[55] Rottig M., Medema M.H., Blin K., Weber T., Rausch C., Kohlbacher O. (2011). NRPSpredictor2-a web server for predicting NRPS adenylation domain specificity. Nucleic Acids Res..

[56] Filloux A., Ramos J.L., Preface (2014). Pseudomonas Methods and Protocols. Methods Mol. Biol..

[57] Swingle B., Bao Z., Markel E., Chambers A., Cartinhour S. (2010). Recombineering using RecTE from *Pseudomonas syringae*. Appl. Environ. Microbiol..

[58] Dennis J.J., Sokol P.A. (1995). Electrotransformation of *Pseudomonas*. Methods Mol. Biol..

[59] Mosberg J.A., Lajoie M.J., Church G.M. (2010). Lambda red recombineering in *Escherichia coli* occurs through a fully single-stranded intermediate. Genetics.

[60] Ringel M.T., Drager G., Bruser T. (2016). PvdN enzyme catalyzes a periplasmic pyoverdine modification. J. Biol. Chem..

