# Supplementary material for: Pyoverdine-Mediated Killing of Caenorhabditis elegans by Pseudomonas syringae MB03 and the Role of Iron in Its Pathogenicity
Source: Int J Mol Sci. 2020 Mar 22;21(6):2198. doi: 10.3390/ijms21062198 (PMC7139650; doi:10.3390/ijms21062198)
Supplement: Supplementary file 1 [file ijms-21-02198-s001.pdf]

International Journal of
Molecular Sciences

MDPI

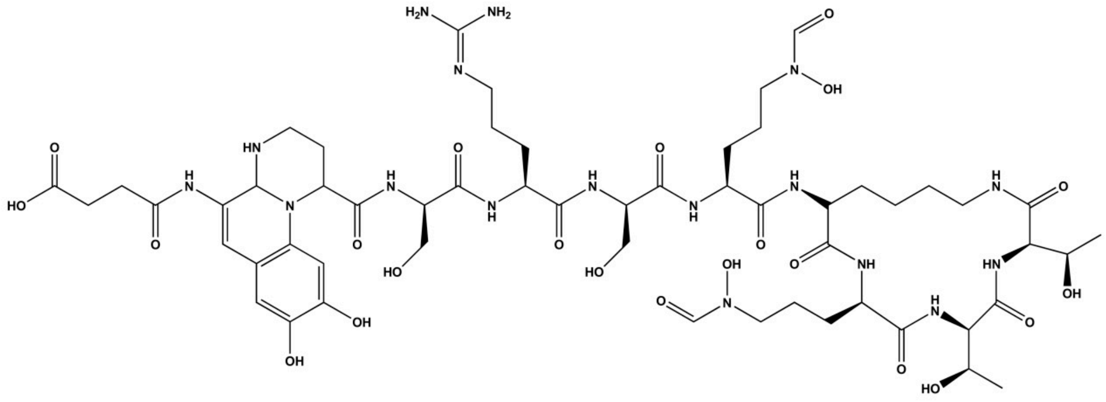

Figure S1. Chemical structure of pyoverdine produced by *P. syringae* MB03.

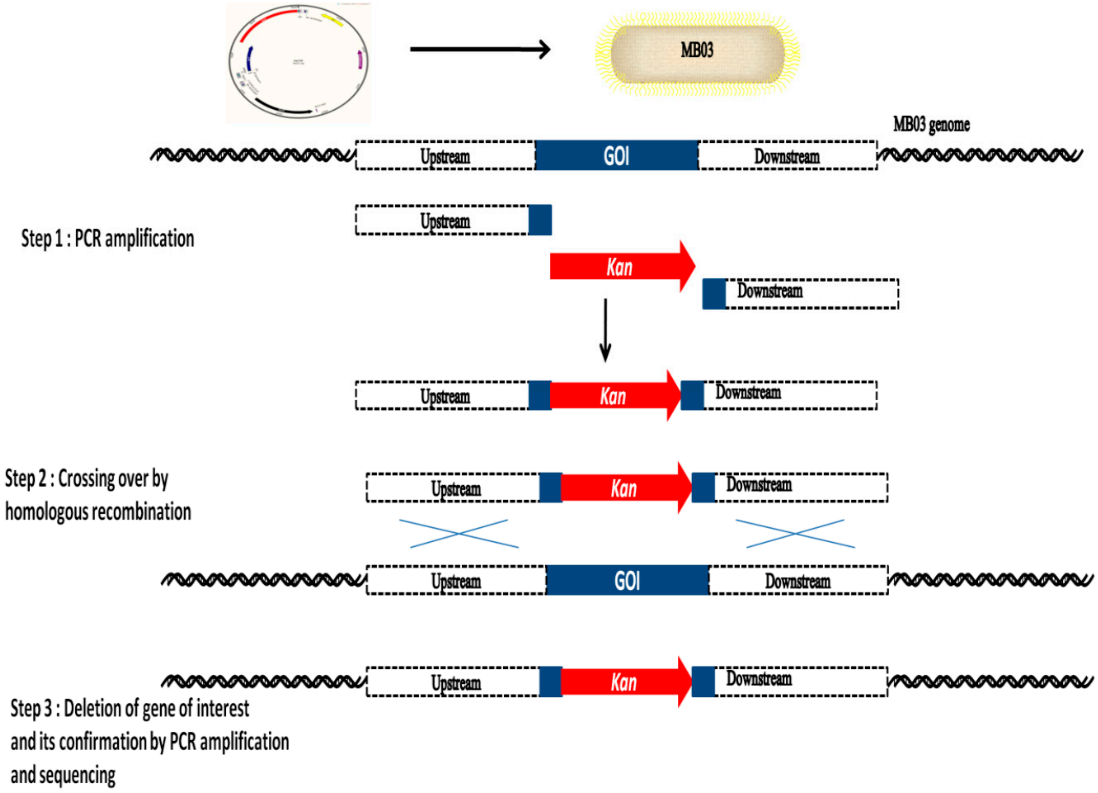

Figure S2. Schematic representation of gene disruption via Rec/TE recombination system.

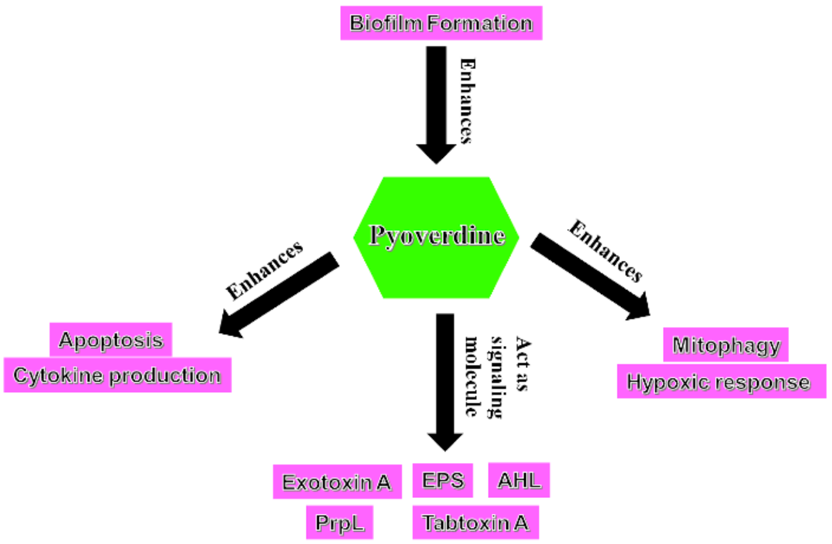

Figure S3. Role of pyoverdine in the pathogenicity. Pyoverdine enhances the innate immunity by induces mitophagy, hypoxic response, apoptosis and cytokine production. Pyoverdine also serves as signaling molecule by stimulating the production of other virulence factors in *P. aeruginosa* such as Exotoxin A and PrpL. Biofilm formation enhances the production of pyoverdine.

Table S1. Summary of *P. aeruginosa* PAO1 and *P. syringae* MB03 pyoverdine genes.

PAO1 Genes	Annotated Function in *P. aeruginosa* PAO1	Orthologs in *P. syringae* MB03
pvdX	Regulatory protein	**Absent**
pvdY	Regulatory protein	**Absent**
fpvR	Anti-sigma factor for *pvdS*	VT47-09085
pvdS	ECF iron sigma 70 factor	VT47_09155
pvdG	Thioesterase	VT47_09160
pvdL	Chromophore peptide synthetase	VT47_09165
pvdH	Aminotransferase	VT47_09170
Pa_2412	MbtH-like protein	VT47_09175
Pa_2403-2406	ABC transporters	VT47_09180-09195
Pa_2407-2410	Hypothetical proteins	VT47_09200-09215
**Absent**	Asparate hydroxylase	VT47_09220
pvdD	Pyoverdine peptide chain-NRPS	VT47_09225-0940
pvdI	Pyoverdine peptide chain-NRPS	VT47_09230
pvdJ	Pyoverdine peptide chain-NRPS	VT47_09235
pvdK	Pyoverdine peptide chain-NRPS	VT47_09240
fpvA	Ferripyoverdine receptor protein	VT47_09245,09250
pvdE	Cyclic peptide ABC transporter	VT47_09255
pvdONMP	Periplasmic proteins probably involve in the maturation of pyoverdine	VT47_09260-09275
pvdRT-Omp	RND/MFP/OMF type-efflux system	VT47_09280-09290
pvdQ	Acylase	VT47_09295
pvdA	L-orinithine hydroxylase	**Absent**
pvdF	N5-hydroxyornithine transformylase	**Absent**
fpvI	ECF sigma factor required for the expression of *fpvA*	**Absent**
